# Dynamic Changes of CD44 Expression from Progenitors to Subpopulations of Astrocytes and Neurons in Developing Cerebellum

**DOI:** 10.1371/journal.pone.0053109

**Published:** 2013-01-04

**Authors:** Masae Naruse, Koji Shibasaki, Shuichi Yokoyama, Masashi Kurachi, Yasuki Ishizaki

**Affiliations:** Department of Molecular and Cellular Neurobiology, Gunma University Graduate School of Medicine, Maebashi, Japan; University of Wuerzburg, Germany

## Abstract

We previously reported that CD44-positive cells were candidates for astrocyte precursor cells in the developing cerebellum, because cells expressing high levels of CD44 selected by fluorescence-activated cell sorting (FACS) gave rise only to astrocytes *in vitro*. However, whether CD44 is a specific cell marker for cerebellar astrocyte precursor cells *in vivo* is unknown. In this study, we used immunohistochemistry, *in situ* hybridization, and FACS to analyze the spatial and temporal expression of CD44 and characterize the CD44-positive cells in the mouse cerebellum during development. CD44 expression was observed not only in astrocyte precursor cells but also in neural stem cells and oligodendrocyte precursor cells (OPCs) at early postnatal stages. CD44 expression in OPCs was shut off during oligodendrocyte differentiation. Interestingly, during development, CD44 expression was limited specifically to Bergmann glia and fibrous astrocytes among three types of astrocytes in cerebellum, and expression in astrocytes was shut off during postnatal development. CD44 expression was also detected in developing Purkinje and granule neurons but was limited to granule neurons in the adult cerebellum. Thus, at early developmental stages of the cerebellum, CD44 was widely expressed in several types of precursor cells, and over the course of development, the expression of CD44 became restricted to granule neurons in the adult.

## Introduction

The cerebellum is composed of distinct layers: the external germinal layer (EGL), the molecular layer (ML), the Purkinje cell layer (PCL), the granule layer (GL), and the white matter (WM) [1]. There are two germinal centers in the embryonic cerebellum. The ventricular zone gives rise to GABAergic neurons and glial lineages, and the rhombic lip gives rise to glutamatergic neurons [2]–[5]. In the postnatal cerebellum, multipotent neural stem cells in the white matter can generate inhibitory interneurons, astrocytes, and oligodendrocytes [6], [7]. There are three types of astrocytes in the murine cerebellar cortex: Bergmann glia in the Purkinje cell layer, fibrous astrocyte in the white matter, and protoplasmic astrocyte in the granule layer [8]. By utilizing this specific characteristic, we can easily identify those astrocytes judging from their morphologies and locations, therefore we focused on developing cerebellum as a good model to examine glial development. However, how these different types of cerebellar astrocytes are generated remains poorly understood. We previously have shown that cells with high CD44 expression (CD44^high^ cells), purified from the large-cell fraction (enriched in glia) of mouse postnatal day 3 (P3) cerebellum, were astrocyte-restricted precursor cells *in vitro* [9].

CD44 is a transmembrane glycoprotein implicated in cell–matrix adhesion and matrix-mediated cell signaling [10]. CD44 is known as a receptor for extracellular components such as hyaluronic acid [11] and osteopontin [12]. CD44 can be cleaved by ADAM (A Disintegrin And Metalloproteinase) protease, matrix metalloproteinase, and γ-secretase, resulting in the release of an extracellular domain of CD44 in soluble form and an intracellular domain of CD44 that functions as a transcription factor in the nucleus [13]–[15]. CD44 is involved in several cellular processes including cell migration, survival, differentiation, and motility [11] and is known as a cancer stem cell marker [16], [17]. CD44 is expressed in glioma in the central nervous system [18], [19]. It is also expressed in astrocyte-lineage cells in a dorsal domain of the rodent embryonic spinal cord [20], [21], Mueller glia-committed retinal progenitor cells [22], and at a low level, in astrocytes in the cortex and spinal cord [23]–[27]. On the other hand, oligodendrocytes express detectable levels of CD44 only in pathological situations [28]. CNP-CD44 transgenic mice with overexpression of CD44 in glial progenitors had decreased oligodendrocyte maturation [20]. These results indicate that CD44 has also important roles in oligodendrocyte differentiation, in addition to its roles in astrocytes. Although we have isolated candidates of astrocyte precursor cells from the developing cerebellum on the basis of their expression of CD44 as described above [9], it is unclear whether CD44 is expressed only in astrocyte-lineage cells in the cerebellum during development.

In this study, we clarified the spatial and temporal expression profiles of CD44 during development of the mouse cerebellum by immunohistochemistry, *in situ* hybridization, and fluorescence-activated cell sorting (FACS).

## Materials and Methods

All experiments were performed in accordance with the Guidelines for Animal Experimentation at Gunma University Graduate School of Medicine and were approved by the Gunma University Ethics Committee. We used more than 3 animals for each experiment to conclude the results.

### Animals

**Figure 1:**
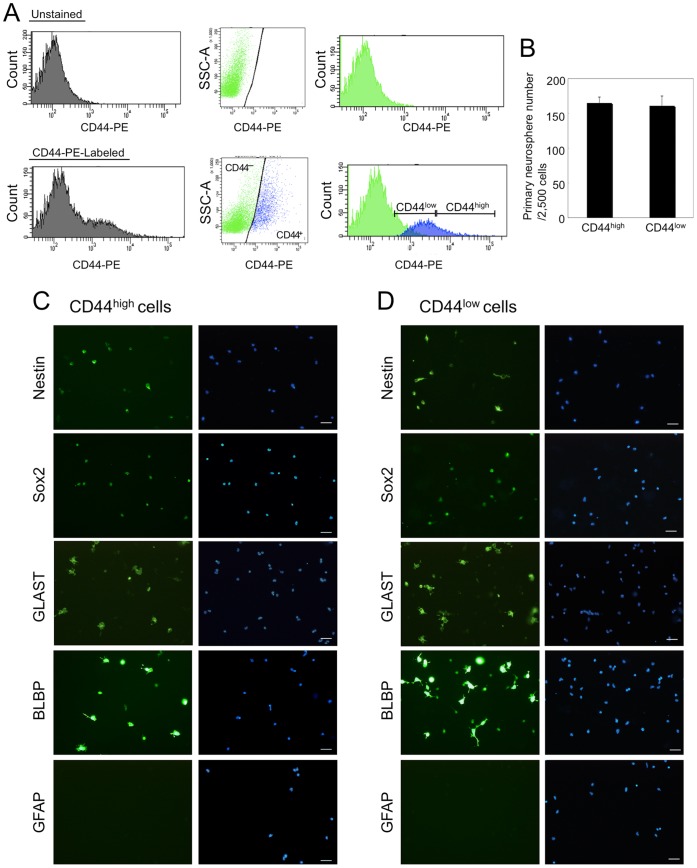
The characterization of CD44^high^ cells and CD44^low^ cells. **A:** FACS plots of CD44 positive cells from glial-enriched cellular fraction of P3 mouse cerebellum. There was overlap in the histogram of cell number vs fluorescence intensity of CD44 antibody staining (CD44–PE) between the CD44-positive population and the CD44-negative population (left histograms). To separate CD44-positive cells from CD44-negative cells, side scatter (SSC) vs. CD44-PE (Dot plots) and the gates to separate the CD44-negative population from the CD44-positive population (reflected in the right histograms) were determined. CD44^high^ cells (about 5% of glial-enriched cellular fraction) and CD44^low^ cells were isolated by FACS. **B:** The number of primary neurospheres. **C&D:** Immunostaining of nestin, Sox2, GLAST, BLBP and GFAP on CD44^high^ cells (**C**) and CD44^low^ cells (**D**) isolated by FACS. Scale bars, 50 µm.

**Figure 2:**
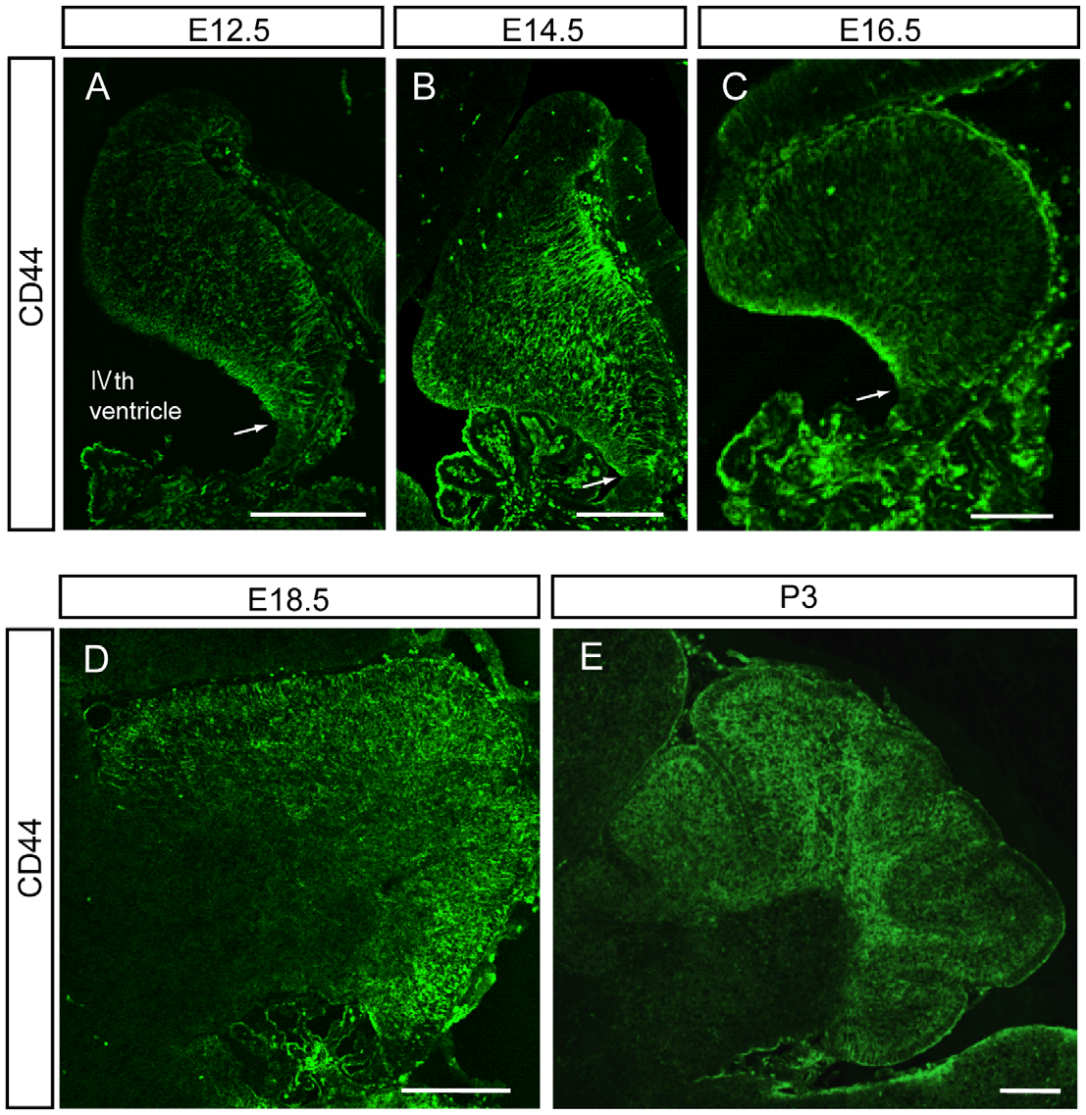
Developmental expression of CD44 in mouse cerebellum. CD44 was detected by Tyramide Signal Amplification methods in brain sections from E12.5 to P3 mice. Representative fluoroscence micrographs are shown. The arrow is placed on the edge of CD44-positive and negative regions. Scale bars, 200 µm.

**Figure 3:**
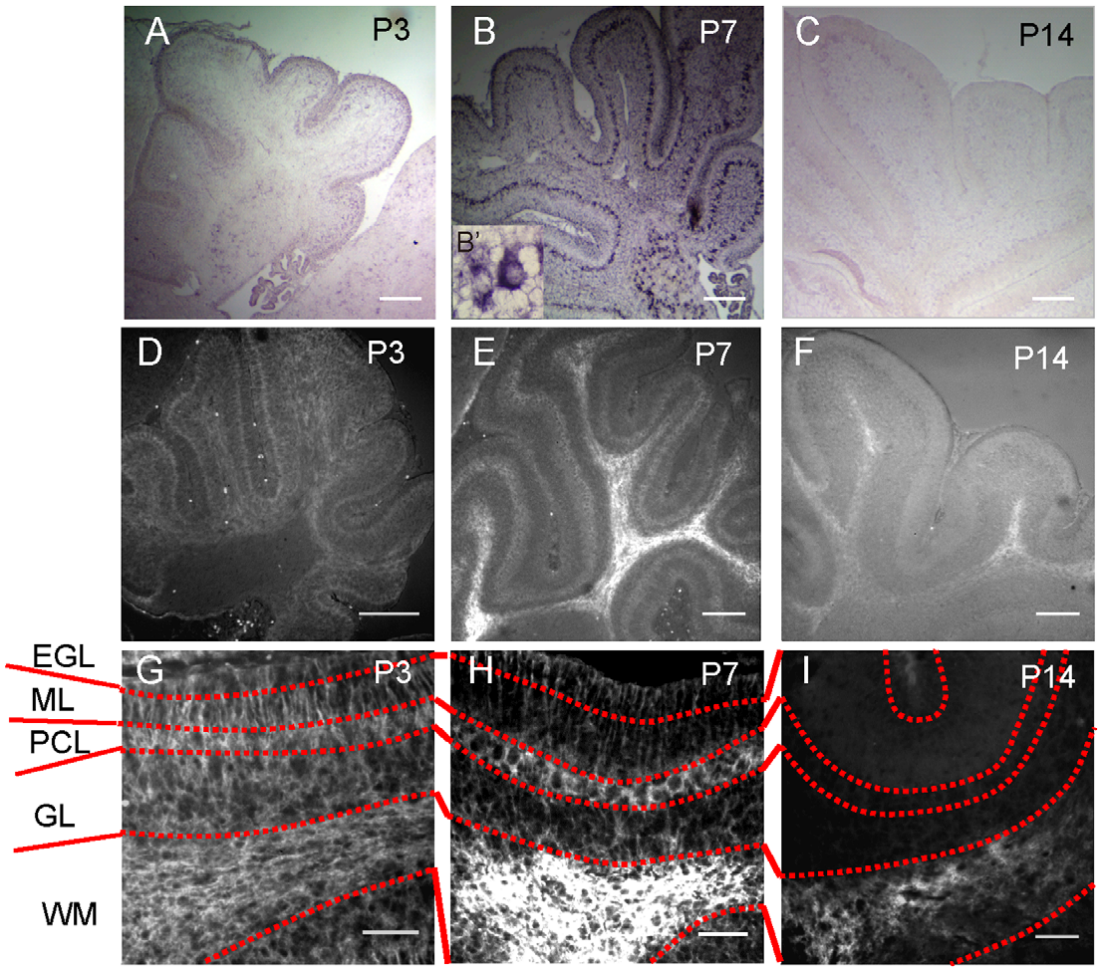
CD44 expression in postnatal mouse cerebellum at P3, P7 and P14. **A–C:** Detection of *CD44* mRNA by *in situ* hybridization. **B’:** High magnification of *CD44* mRNA signals in PCL at P7. **D–E:** Detection of CD44 by PE-labeled anti-CD44 antibody. **G–I:** High magnification of figures D–E. Scale bars, 200 µm (A–E) or 50 µm (G–I).

**Figure 4:**
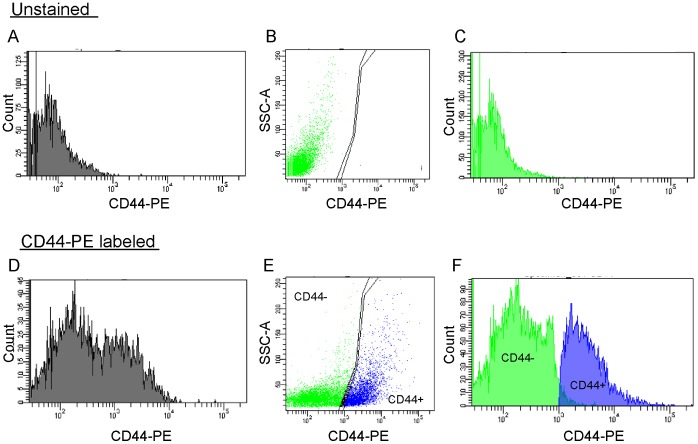
Representative FACS plots of CD44 positive cells from P3 mouse cerebellum. **A–C:** Unstained sample. **D–F:** CD44-stained sample. The CD44-positive population and CD44-negative population overlapped in the histogram of cell number vs. fluorescence intensity from CD44 antibody staining (CD44-PE) (A and C). To separate CD44-positive cells from CD44-negative cells clearly, dot plots depicted SSC vs. CD44-PE (B and D), and gates to separate the CD44-negative population from the CD44-positive population were determined. The gate information was reflected again by histogram (C and F). The CD44-positive cell population shown in E was isolated by FACS.

**Figure 5:**
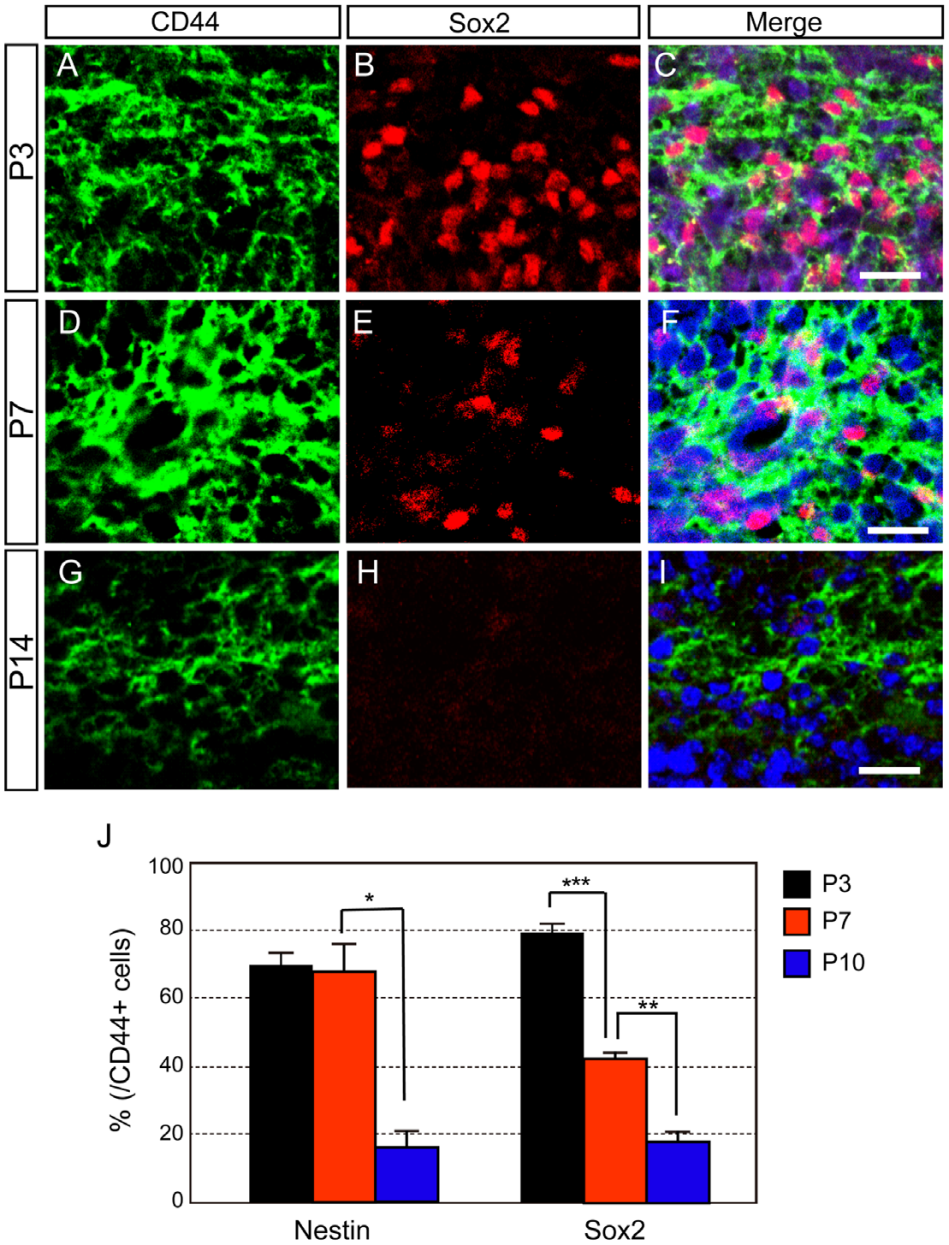
CD44 expression in neural stem/progenitor cells during postnatal development. **A–I:** Double immunostaining of CD44 and Sox2 in the cerebellum at P3 (A–C), P7 (D–F) and P14 (G–I). Nucleus was counterstained with TO-PRO-3 (blue). **J:** Quantitative analysis of the number of CD44-positive neural stem/progenitor cells by FACS at P3, P7 and P10. *p<0.05, **p<0.005, ***p<0.001. Scale bars, 20 µm.

**Figure 6:**
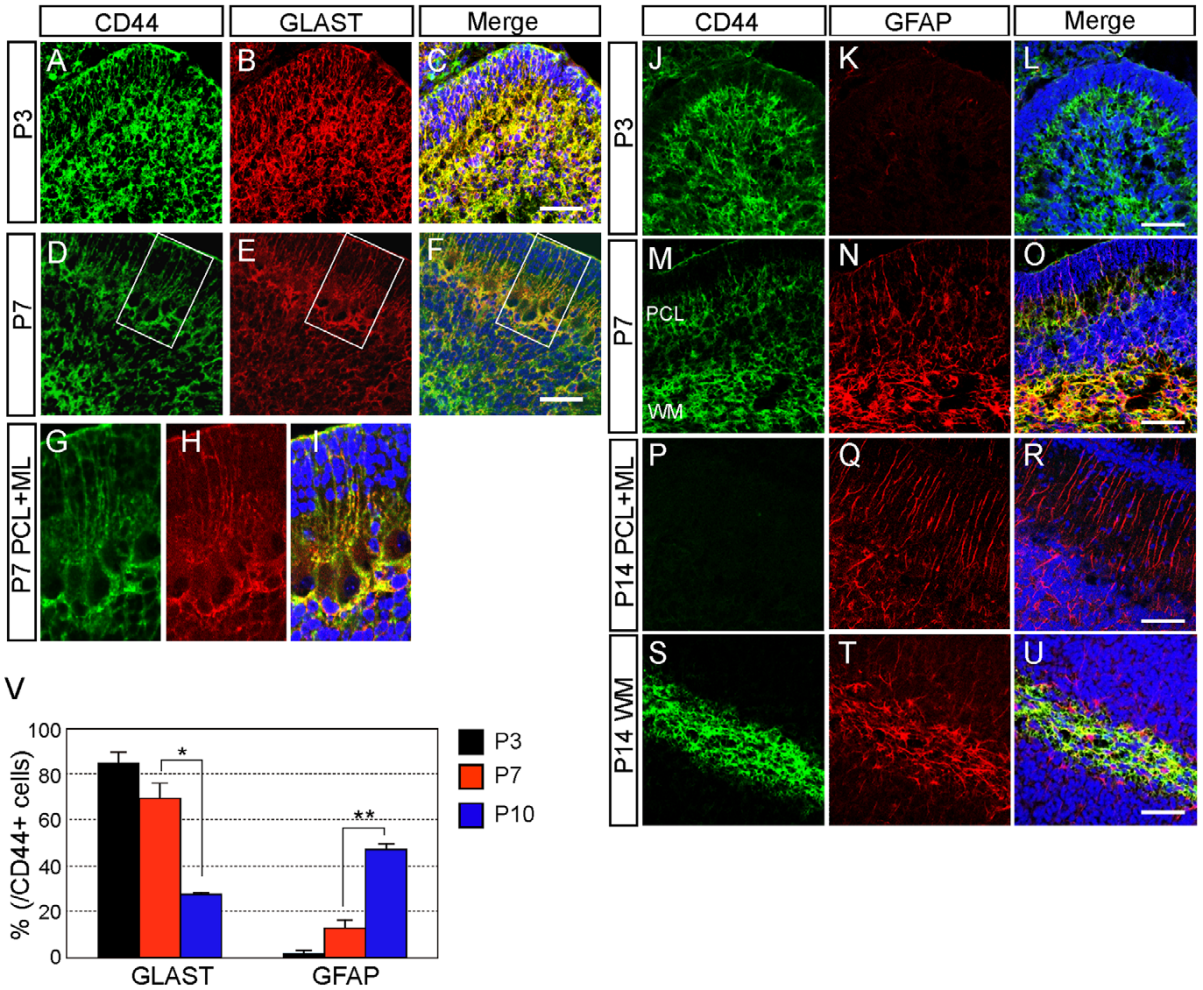
CD44 expression in astrocyte-lineage cells during postnatal development. A–I: Double immunostaining of CD44 and GLAST in the cerebellum at P3 (A–C) and P7 (D–F). **G–I:** High magnification of D–F. **J–U:** Double immunostaining of CD44 and GFAP in the mouse cerebellum at P3 (J–L) and P7 (M–O), and at P14 in the Purkinje cell layer (P–R) and white matter (S–U). Nucleus was counterstained with TO-PRO-3 (blue). **V:** Quantitative analysis of the number of CD44-positive astrocyte-lineage cells by FACS at P3, P7 and P10. *p<0.05, **p<0.005. Scale bars, 50 µm.

**Figure 7:**
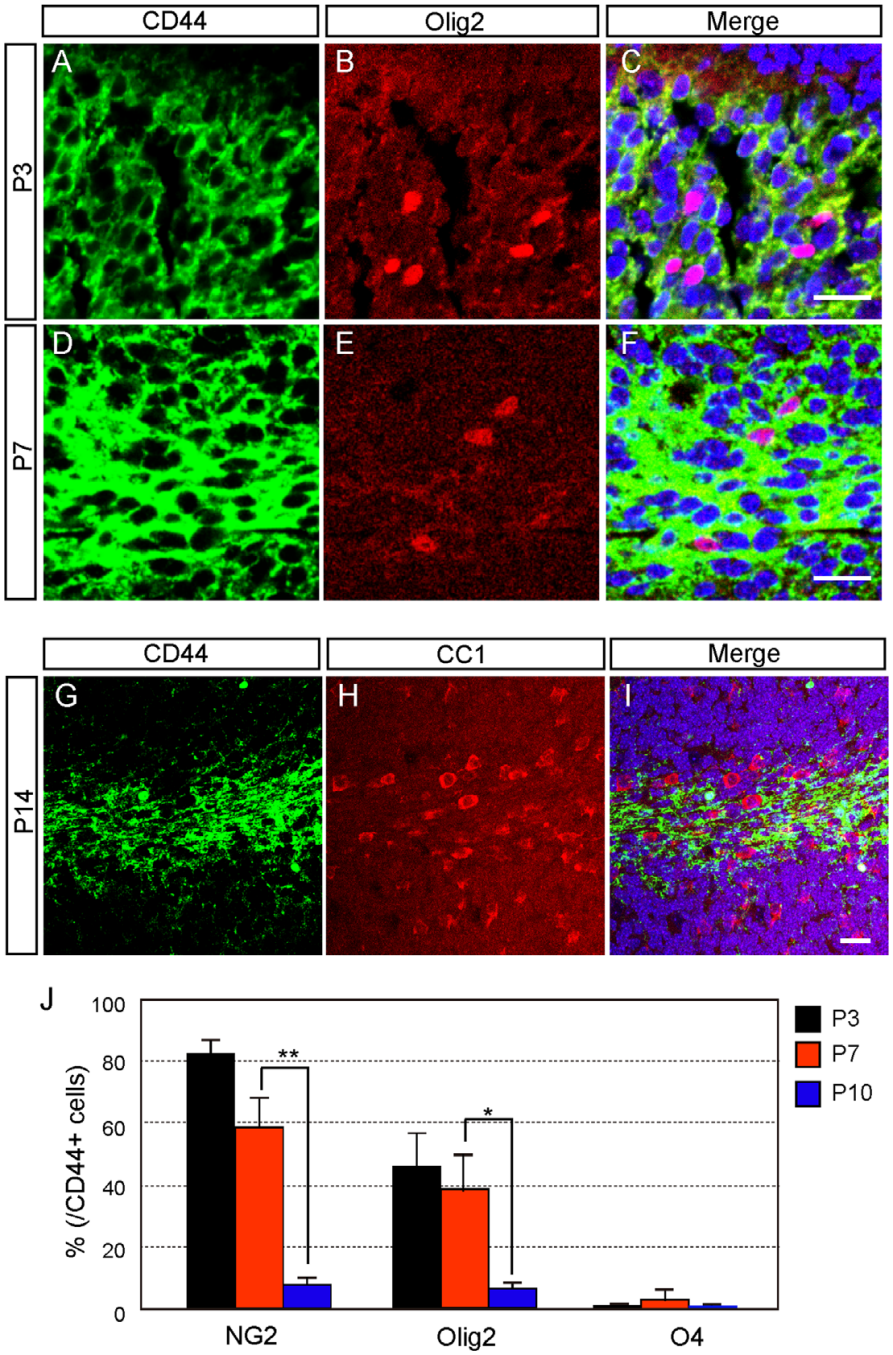
CD44 expression in oligodendrocyte-lineage cells during postnatal development. **A–F:** Double immunostaining of CD44 and Olig2 in the cerebellum at P3 (A–C) and P7 (D–F). **G–I:** Double immunostaining of CD44 and CC1 at P14. Nucleus was counterstained with TO-PRO-3 (blue). **J:** Quantitative analysis of the number of CD44-positive oligodendrocyte-lineage cells by FACS at P3, P7 and P10. *p<0.05, **p<0.005. Scale bars, 20 µm.

**Figure 8:**
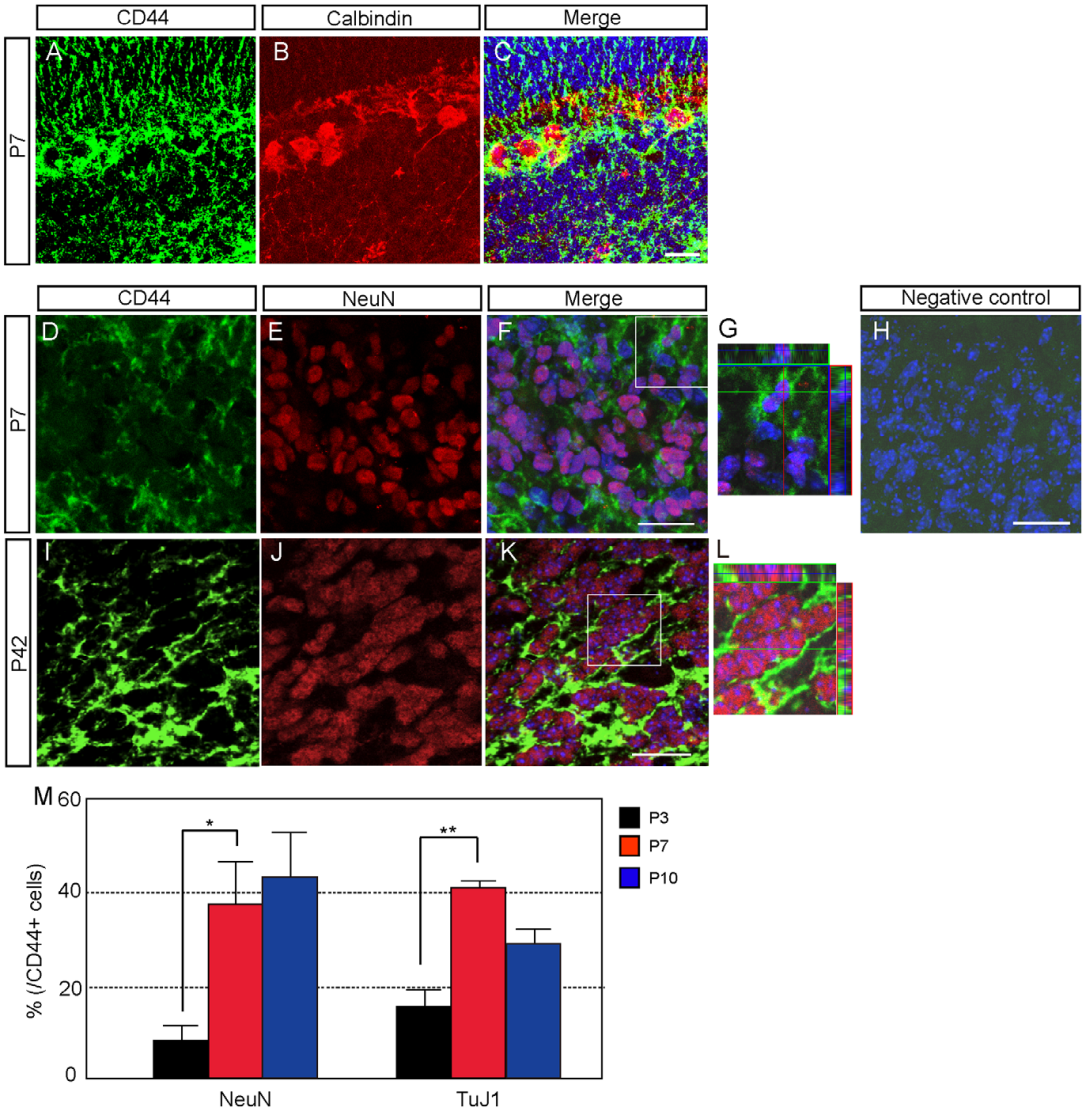
CD44 expression in neuron-lineage cells during postnatal development. A–C: Double immunostaining of CD44 and calbindin in the cerebellum at P7. **D–L:** Double immunostaining of CD44 and NeuN at P7 (D–H) and P42 (I–L). **H:** Negative controle. **G&L:** High magnification of F&K. Nucleus was counterstained with TO-PRO-3 (blue). **J:** Quantitative analysis of the number of CD44-positive neuron-lineage cells by FACS at P3, P7 and P10. *p<0.05, **p<0.005. Scale bars, 20 µm.

C57BL6/NCr (SLC, Japan) (Fig. 1) or ICR strain mice (SLC, Japan) (Fig. 2–8) were used throughout the studies. Embryos were collected at E12.5, E14.5, E16.5 and E18.5, and pups were collected at P3, P7, P10 and P14. Embryos (E14.5–E18.5), pups, and adults (P42) were perfused transcardially with phosphate buffered saline (PBS) followed by 4% paraformaldehyde (PFA) in PBS under deep anesthesia. Brains were further fixed in the same fixative over night at 4°C, and then immersed in PBS containing 20% sucrose. Brains fixed with 4% PFA were cut sagittally with a cryostat at a thickness of 18 µm.

### Immunostaining with Phycoerythrin-conjugated Anti-CD44 Antibody

The brain sections were washed with PBS, incubated for 30 min in TNB buffer (0.1 M Tris-HCl, 0.15 M NaCl, 0.5% Blocking regent), then incubated with phycoerythrin (PE)-conjugated rat anti-CD44 antibody (BD Biosciences, Clone name is IM7; diluted 1∶200 in TNB buffer) overnight. After washing with PBS, the sections were examined with fluorescence microscopy (Axiovert 135, Zeiss, Germany).

### Double Immunostaining for CD44 and Cellular Markers

Fixed brain sections were incubated in blocking buffer (3% BSA/PBS with 0.3% Triton X-100) and then were incubated with CD44 antibody (IM7, hybridoma supernatant; American Type Culture Collection; diluted 1∶1000 in TNB buffer) for 2 hr. The sections were washed with PBS, incubated with biotin-conjugated anti-rat antibody, washed again with PBS, and incubated with Streptavidin-HRP. The CD44 signal was detected by using Tyramide Signal Amplification methods (TSA Plus Fluorescein Evaluation Kit; PerkinElmer, Waltham, MA). The sections were then incubated with primary antibodies to cellular markers for 2 hr at room temperature. The primary antibodies used included antibodies directed against GLAST (1∶1000; Frontier Science, Hokkaido, Japan), GFAP (1∶400; Millipore-Chemicon), Sox2 (1∶100; Cell Signaling Technology, Danvers, MA), Olig2 (1∶20; IBL, Takasaki, Japan), CC1 (1∶400; Calbiochem, San Diego, CA), Calbindin (1∶100; Cell Signaling Technology) and NeuN (1∶100; Millipore-Chemicon). The sections were washed with PBS and subsequently incubated with rhodamine-conjugated secondary antibodies. TO-PRO-3 (Invitrogen-Molecular Probes; 2.5 µM) was used as a counterstain. Sections were mounted with Vectashield mounting medium (Vector Laboratories, Burlingame, California) and examined with confocal laser scanning microscopy (LSM 510 Meta, Zeiss, Germany).

### CD44^high^ and CD44^low^ Cell Isolation by FACS and Neurosphere Assay

CD44^high^ cells and CD44^low^ cells were isolated as previously described [9]. C57BL6/NCr mouse cerebellum at P3 was cut into small pieces and incubated at 37°C for 30 min in papain solution (Dulbecco**’**s phosphate-buffered saline (DPBS) containing 16.5 U/ml papain, 200 µg/ml L-cysteine, and 0.008% deoxyribonuclease). The tissue was rinsed in DPBS containing 1.5 mg/ml bovine serum albumin (BSA) and 0.008% deoxyribonuclease and triturated in the same solution. The cells were centrifuged at 1,000 rpm for 10 min at room temperature and suspended in DPBS containing 10 mg/ml BSA and centrifuged again. The tissue was then resuspended in washing buffer (DPBS containing 0.02% BSA and 5 µg/ml insulin) and passed through a cell strainer, and centrifuged again. The cell suspension was loaded onto a step gradient of 35% and 60% Percoll (GE Healthcare UK Ltd., Little Chalfont, Buckinghamshire, UK) and centrifuged at 3,000 rpm for 20 min at room temperature. The large cell fraction was recovered from the 0%:35% interface, washed twice in washing buffer, and was used for cell isolation by FACS. The cells were suspended in 2%BSA/PBS at a density of 2×10^7^ cells/ml and labeled with PE-conjugated anti-CD44 antibody for 30 min in the dark on ice. The cells were then centrifuged at 1,000 rpm for 10 min and suspended in 2%BSA/PBS at 5×10^6^ cells/ml. To remove dead cells, 7-amino-actinomycin D (BD Biosciences) was added to the cell suspension (20 µl/10^6^ cells). Cells were sorted by using a FACSAria 2 flow cytometer (BD Biosciences). There was overlap in the histogram of cell number vs fluorescence intensity of CD44 antibody staining (CD44-PE) between the CD44-positive population and the CD44-negative population (Fig. 1A left histograms). To separate CD44-positive cells from CD44-negative cells, side scatter (SSC) vs. CD44-PE (Fig. 1A Dot plots) and the gates to separate the CD44-negative population from the CD44-positive population (reflected in the Fig. 1A right histograms) were determined. CD44^high^ cells (about 5% of glial-enriched cellular fraction) and CD44^low^ cells isolated by FACS were used for immunostaining and Neurosphere assay. CD44^high^ cells and CD44^low^ cells were cultured at 5 cells/µl in a 24-well plate (Falcon) in 500 µl of serum free media containing 10 ng/ml basic fibroblast growth factor (FGF2; Sigma) and 2 µg/ml heparin (Sigma). After 7 days *in vitro*, numbers of floating sphere colonies (neurospheres) possessing a diameter of greater than 0.1 mm were counted.

### CD44-positive Cell Isolation from Whole Cerebellum by FACS and Immunostaining

The cell isolation methods were modified from a previous report [9]. Cerebella from P3-P10 mice (ICR) were cut into small pieces and incubated at 37°C for 10 min in trypsin solution (0.05% trypsin, 0.2 mM EDTA). The tissue was rinsed in DPBS (Dulbecco’s phosphate-buffered saline) containing 0.25 mg/ml trypsin inhibitor, 1.5 mg/ml BSA and 0.008% deoxyribonuclease and triturated in the same solution, centrifuged at 1,000 rpm for 10 min at room temperature, resuspended in DPBS containing 10 mg/ml BSA, and centrifuged again. The cells were then resuspended in washing buffer (DPBS containing 0.02% BSA and 5 µg/ml insulin) and passed through a cell strainer (BD Falcon, Franklin Lakes, New Jersey), and centrifuged again, and was used for cell isolation by FACS. CD44-positive cells were plated on 12-well glass slides (Matsunami, Osaka, Japan) precoated with 1% Matrigel (BD Biosciences, Billerica, Massachusetts) and 2 h later were fixed with 4% PFA for immunostaining. The primary antibodies used for cell staining included antibodies against GLAST, GFAP, Sox2, Nestin (1∶100; BD Biosciences), Olig2, O4 (1∶100), NeuN, and β-tubulin class III (1∶200; COVANCE, Berkeley, CA).

### Detection of Cell Proliferation

To identify cells that were in the S phase of the cell cycle in postnatal mice, a subcutaneous (P3 and P7) or intraperitoneal (P10 and P14) injection of bromodeoxyuridine (BrdU; 50 µg/g body weight) was administered for incorporation into newly synthesized DNA. At 2 hr after BrdU injection, pups were perfused transcardially with 4% paraformaldehyde in PBS under deep anesthesia. Brains were further fixed in the same fixative over night at 4°C, and then immersed in PBS containing 20% sucrose. Sagittal sections on glass slides were treated with 2N HCl for 30 min. Following incubation with blocking buffer, the sections were incubated overnight with a mouse anti-BrdU antibody (1∶1000; Pharmingen, San Diego, CA) at 4°C, washed with PBS, and incubated for 1 hr with anti-mouse IgG conjugated to rhodamine.

### In Situ Hybridization

Digoxigenin-labeled antisense/sense probes were used for *in situ* hybridization as previously described [29]. A fragment of mouse CD44 cDNA was obtained by PCR using the primers 5′- CGGAATTCCCGCTACGCAGGTGTATTCC -3′ and 5′- GCTCTAGATAATGGCGTAGGGCACTACAC -3′ (Genbank accession number, NM_009851) [30] and subcloned into the *Eco*RI and *Xba*I sites of pBluescriptSKII(+). After linearizing the plasmid (antisense: *Eco*RI, sense: *Xba*I), digoxigenin-labeled antisense/sense probes were synthesized by RNA polymerase (antisense: T3 RNA polymerase, sense: T7 RNA polymerase). mRNA in cryosectioned tissue (14 µm thickness) was detected with alkaline phosphatase conjugated anti-digoxigenin antibody (Roche) and nitroblue tetrazolium/5-bromo-4-chloro-3′-indolyl phosphate.

### Statistical Analysis

Results are presented as the mean ± SEM. Student’s t-test was used to determine the significance of differences between groups.

## Results

Previously, we have identified cerebellar astrocyte precursor cells. CD44^high^ cells isolated from glial-enriched cellular fraction of P3 mouse cerebellum by FACS were positive for astrocyte-lineage markers (BLBP, GLAST) and the neural stem cell marker (nestin) but were negative for the mature astrocyte marker (GFAP), the immature oligodendrocyte marker (O4) or the neuronal marker (Tuj1). We concluded that these CD44^high^ cells were astrocyte precursor cells because they produced no neurospheres, and gave rise only to astrocytes in the absence of any signaling molecule *in vitro.* [9]. However, we have not characterized CD44^low^ cells. To examine whether only CD44^high^ cells are astrocyte precursor cells or not, we compared the ability of neurosphere formation and the expression of cell-type specific markers between CD44^high^ cells and CD44^low^ cells. CD44^high^ cells and CD44^low^ cells were collected from glial-enriched cellular fraction of P3 mouse cerebellum by the same methods with previous report (Fig. 1A) [9]. Both of CD44^high^ cells and CD44^low^ cells yielded neurospheres under FGF-2 and heparin (Fig. 1B). In our previous report, CD44^high^ cells had been cultured with only FGF-2 (not with heparin), therefore CD44^high^ cells might fail to form neurospheres [9]. Most of CD44^low^ cells expressed nestin, Sox2, GLAST and BLBP as same as CD44^high^ cells (Fig. 1C and 1D). On the other hand, GFAP, O4, and Tuj1 were less expressed in both CD44^high^ cells and CD44^low^ cells (Fig. 1C and 1D, data not shown). This result suggests that CD44^high^ cells do not have a specific character as astrocyte precursors among total CD44-positive cells. The result of neurosphere assay suggested that CD44-positive cells of P3 cerebellum certainly contain neural stem cells. This means we need more careful analysis to determine whether CD44 expression is restricted only to astrocyte-lineage cells or not. Here, we focused on the expression profile of CD44 during cerebellar development in order to determine whether CD44 expression is restricted to astrocyte-lineage cells. CD44 expression was detected as early as E12.5 in the developing mouse cerebellum (Fig. 2A). The CD44 signal was localized near the ventricular zone of the IVth ventricle, but not at the rhombic lip (Fig. 2A, the arrow is placed on the edge of CD44-positive and negative regions). After this stage, the expression of CD44 expanded throughout the cerebellum during embryonic development (from E14.5 to E18.5, Fig. 2B-2D). To further analyze postnatal CD44 expression, we performed *in situ* hybridization and immunohistochemistry of CD44 at P3, P7 and P14. CD44 expression was observed in all layers of the cerebellum at P3 (Fig. 2E, 3A, 3D and 3G); however, the expression of CD44 was mainly restricted to the PCL and WM at P7 (Fig. 3B, 3E and 3H). CD44 is a cell surface protein, the expression of CD44 detected by immunostaining was observed on the cell body in PCL and on the process in ML, although *CD44* mRNA was detected around nucleus of Bergmann glia or Purkinje neuron in PCL (Fig. 3B’). Only a very weak signal was detected in the EGL, ML and GL at P7 (Fig. 3E and 3H). Finally, the strong signal was detected only in the WM at P14 (Fig. 3C, 3F and 3I). Very weak signals were still detected in the GL at P14 (Fig. 3C, 3F and 3I). These results indicate that CD44 expression changes depending on the developmental stage of the cerebellum. *In situ* hybridization probe for CD44 (targeting the regular last four exons) and anti-CD44 antibody (IM7) recognize all isoforms of CD44, although there are many splice isoforms of CD44 [31].

Next, we analyzed which cell types express CD44. Since CD44 is a cell surface protein, it was very difficult to count the numbers of CD44-positive cells with several cell markers by immunohistochemical analysis. Therefore, we mainly used FACS analysis to quantify cell marker expression by CD44-positive cells at various developmental stages. All CD44-positive cells were isolated from whole cerebellum (not from glial-enriched cellular fraction) (Fig. 4). CD44 immunostaining with a direct method using PE-conjugated anti-CD44 antibody (Fig. 3) and with the Tyramide Signal Amplification method (Fig. 2E and Fig. 5–8) provided similar CD44 expression patterns. First, we examined CD44 expression in neural stem cells, which are located in the WM of the postnatal cerebellum [6]. Analysis of *in vitro* cultures and genetic examination has implicated Sox2-positive cells include neural stem cells [32]. The majority of CD44-positive cells were thought to be neural progenitor cells at P3, since over 80% of CD44-positive cells were identified as Sox2-positive cells by immunohistochemical and FACS analysis (Fig. 5A–C and 5J). The percentage of CD44-positive cells that expressed Sox2 had decreased by P7 (Fig. 5D–F and 5J) and was less than 20% at P14 (Fig. 5G–I and 5J). Coexpression of CD44 and nestin showed a similar developmental pattern (Fig. 5J). These results indicate that CD44 is expressed in neural stem/progenitor cells at early postnatal stages and suggest that the number of CD44-expressing neural stem/progenitor cells decreases during cerebellar development. The reduction of neural stem/progenitor cell number in postnatal cerebellum (Fig. 5B, 5E and 5H) was supported by previous report, which revealed dividing cells and nestin-positive cells decreased in rat postnatal cerebellum during development [33].

Next, we focused on astrocyte-lineage cells. Consistent with our previous report, most of the CD44-positive cells in mouse cerebellum co-expressed GLAST at P3 (Fig. 6A–C). It is difficult, however, to distinguish between immature astrocytes and neural stem/progenitor cells in P3 cerebellum, as many Sox2-positive cells also expressed GLAST (Supporting Figure S1A–D). The CD44/GLAST-positive cells were detected only in the PCL and WM at P7 (Fig. 6D–F). The CD44/GLAST double-positive cells that had their cell bodies in the PCL extended radial processes to the pial surface, showing these cells as immature Bergmann glia (Fig. 6G–I, Supporting Figure S2, asterisk showed the cell body of CD44/GLAST double-positive Bergmann glia), whereas the CD44/GLAST double-positive cells located in the WM were immature fibrous astrocytes (Fig. 6D–F). FACS-sorted CD44-positive cells (Fig. 4) were immunostained for GLAST and GFAP. The percentage of CD44-positive cells that expressed GLAST was high at P3 and gradually decreased during postnatal development (Fig. 6V). In contrast, CD44/GFAP-positive cells were rarely observed at P3 (Fig. 6J–L). CD44/GFAP-positive cells were observed in the PCL and WM at P7 (Fig. 6M–O). CD44/GFAP-positive cells were detected only in the WM, and not in the PCL, at P14 (Fig. 6P–U). These results indicate that only fibrous astrocytes expressed CD44 after astrocyte maturation.

To determine whether cells of non-astrocytic lineage express CD44 or not, we examined oligodendrocyte-lineage cells. Approximately half of the CD44-positive cells coexpressed Olig2, which is a marker for oligodendrocyte precursor cells (OPCs), at P3 and P7 (Fig. 7A–F and 7J). In addition, the number of CD44-positive OPCs decreased during development (Fig. 7J). The percentage of CD44-positive cells that expressed NG2 (an OPC marker) was similar to that of CD44/Olig2 double-positive cells (Fig. 7J). Because some of NG2-positive cells have GLAST expression at P3 (Supporting Figure S1E–J), immature astrocytes and OPCs cannot be distinguished completely at P3 cerebellum. However, none of the CD44-positive cells were positive for CC1 (an oligodendrocyte marker) at P14 (Fig. 7G–I). CD44-positive cells that expressed O4 (an immature oligodendrocyte marker) were rarely detected throughout postnatal development (Fig. 7J). These results indicate that CD44 was transiently expressed in OPCs, and its expression was shut off during oligodendrocyte differentiation.

We further analyzed CD44 expression in the neuronal lineage. Few cerebellar neurons expressed CD44 at P3 (Fig. 8M); however, most of the immature Purkinje neurons expressed CD44 at P7 (Fig. 8A–C). Most of Immature granule neurons did not express CD44 (Fig. 8D–F), but some immature granule neurons expressed CD44 (Fig. 8D–F and 8G). At P7, about 40% of CD44-positive cells isolated by FACS expressed NeuN (Fig. 8M). As it is possible to isolate the cells expressing CD44 only weakly from the CD44-negative cells by FACS, there is a possibility that neurons expressing CD44 only weakly were detected by FACS but not by immunostaining. This datum also supported that a part of immature granule neurons might express CD44 weakly. In the adult, most of the granule neurons in the GL expressed CD44 (Fig. 8I–L). Interestingly, whereas immature granule neurons less or weakly expressed CD44 (Fig. 8D–G), mature neurons strongly expressed CD44 (Fig. 8I–L), suggesting that CD44 might be related to maturation and/or functions of these neurons.

## Discussion

In this study, we revealed dynamic changes of CD44 expression in the mouse cerebellum during development. At E12, CD44 was observed in the ventricular zone of the IVth ventricle, but not in the rhombic lip. The expression of CD44 expanded into the entire cerebellum between E14 and P3 (Fig. 2), after which expression became restricted to specific regions: strong expression of CD44 was observed in the PCL and WM at P7, and in the WM only at P14 (Fig. 3). Interestingly, three types of astrocytes showed different CD44 expression patterns (Fig. 6). GFAP-positive astrocytes appearing in the GL around P7 are protoplasmic astrocytes, and these astrocytes did not express CD44. CD44 expression was observed in immature Bergmann glia at P3 and P7. However, the expression of CD44 in Bergmann glia was never observed after P14. Fibrous astrocytes in the WM expressed CD44 from P3 to P14. The expression of CD44 in the WM (Fig. 6) decreased at P14, and it was lost by the adult stage. Thus, CD44 expression in astrocytes was transiently restricted to immature Bergmann glia and fibrous astrocytes during development. We previously isolated astrocyte precursor cells from P3 cerebellum by selecting cells that express high levels of CD44 [9]. The characteristics of CD44^high^ cells, however, could not be discerned from those of CD44^low^ cells sorted by FACS (Fig. 1) and in the sections from developing cerebellum (Fig. 6), and a part of CD44^high^ cells yield neurospheres (Fig. 1), raising the question whether CD44 expression was restricted to only astrocytic lineages. Therefore, we examined CD44 expression in other cell types.

Remarkably, many CD44-positive cells co-expressed Sox2 and nestin, markers for neural stem cells and progenitor cells (Fig. 5). The majority of CD44-positive cells were thought to be neural progenitor cells at P3, and the percentage of CD44-positive cells that were identified as neural progenitor cells decreased during development. Consistent with the expression of CD44 by progenitors, CD44-positive cells proliferated, and the number of proliferative CD44-positive cells (CD44/BrdU double-positive cells) decreased during development (Supporting Figure S3). CD44 has been reported to be associated with aggressive tumor growth and proliferation [34], [35]. Hence, CD44 might be an important molecule for proliferation of cerebellar progenitor cells.

Notably, OPCs also expressed CD44, and the timing of CD44 expression in these cells was restricted. CD44 expression disappeared during oligodendrocyte maturation (Fig. 7). CD44 expression was also observed in subpopulations of immature neurons (Fig. 8). CD44 was expressed in immature Purkinje neurons at P7, after which expression of CD44 disappeared from the PCL (Fig. 8). In contrast, granule neurons expressed CD44 more strongly in the adult than during developmental stages (Fig. 8). Thus, the expression of CD44 was restricted to granule neurons and had a regulated time course during development.

CD44 is expressed by hematopoietic cells, including stem and progenitor cells [10], [11], [36] and cancer stem cells [16]. In the central nervous system, it was reported that a subset of CD44^+^/CD90^+^ cells in the cortex was capable of neurosphere initiation [37]. In this study, we indicated that CD44 was expressed in the ventricular zone of the cerebellum at early embryonic stages (Fig. 2) and in Sox2-positive cells at early postnatal stages (Fig. 5). A part of CD44-positive cells yield neurospheres (Fig. 1). Based on these results, we hypothesize that CD44 is expressed in neural progenitor cells, including neural stem cells, in both the embryonic ventricular zone and postnatal WM. Therefore, further careful analysis will be required to determine whether CD44-positive cells give rise to only astrocytes, or give rise to not only astrocytes but also other types of cells *in vivo*.

In addition to fibrous astrocytes, we found that CD44 was also transiently expressed in Bergmann glia (Fig. 6). Hyaluronic acid, which is one of the ligands of CD44, is strongly expressed in the WM at P7 and P15 in the cerebellum [38]. The interaction of hyaluronic acid and CD44 in astrocytes stimulates Rac1 signaling, leading to cytoskeletal effects and cell migration [39], suggesting that Bergmann glia and fibrous astrocytes might require CD44 for their migration. Recently, it was reported that cleavage of CD44 generates a CD44 intracellular domain (ICD) that functions as a transcription factor [15]. This transcriptional cascade might be important for cerebellar development. On the other hand, differentiating protoplasmic astrocytes were CD44 negative, although most of astrocyte precursors seemed to be CD44-positive (Fig. 6). Thus there is a possibility that CD44 expression must be shut off in astrocyte precursor cells, if they differentiate into protoplasmic astrocytes. Alternatively, generation of protoplasmic astrocytes might be independent from CD44-positive astrocyte precursors. So far we cannot address these questions, since we have never done the lineage analysis. Fine lineage analysis and further functional analysis is necessary to determine the roles of CD44 in the developing cerebellum.

The expression of CD44 in OPCs was transient and disappeared from immature oligodendrocytes (Fig. 7). The peak of OPC proliferation in cerebellum is around P4, and the number of OPCs increases until P7 [40]. Mature oligodendrocytes, identified by expression of CC1 and MBP, first appear at P6 [40]. In light of the developmental time course of OPCs, the reduction in the number of CD44-positive cells expressing OPC during development suggested that CD44 expression disappeared from OPCs. Thus, the elimination of CD44 from OPCs may have synchronized the switching from proliferation to differentiation of OPCs, suggesting that CD44 inhibits oligodendrocyte differentiation. Consistent with this idea, it was reported that CNP-CD44 transgenic mice with overexpression of CD44 in glial progenitors had decreased oligodendrocyte maturation and increased number of astrocytes in the cortex [20]. In addition, hyaluronic acid accumulated in inflammatory demyelinating lesions and inhibited OPC maturation *in vitro* [41]. It has been hypothesized that CD44 elimination in OPCs might be essential for oligodendrocyte differentiation. We, for the first time, revealed that CD44 is expressed in OPCs for a very short time (Fig. 7); the method we used might be a good tool for the analysis of how OPCs mature in the developing cerebellum.

Strong CD44 expression was observed in immature Purkinje neurons (Fig. 8), and CD44 disappeared from Purkinje neurons after their maturation, similar to its disappearance from Bergmann glia and fibrous astrocytes. The rhombic lip, which generates granule neurons, had less expression of CD44, and granule neurons in the GL at P7 expressed CD44 very weakly. However, granule neurons at the adult stage showed strong expression of CD44, consistent with a previous report of CD44 expression in subsets of NeuN-positive neuronal-lineage cells at the adult stage [30]. These results suggest that CD44 might have different roles in Purkinje neurons and granule neurons. It is possible that CD44 might regulate the development of immature Purkinje neurons and circuitry functions of granule neurons. Granule neurons express CD44 strongly in the adult, so CD44 might be required for glutamatergic transmissions. Although little is known about the role of CD44 in neuronal functions, it was reported that CD44 limited axonal sprouting induced by kainic acid in the hippocampus [42].

In this study, we show that the expression of CD44 was widespread in undifferentiated progenitor cells at embryonic stages and restricted to subpopulations of astrocytes and neurons. Finally, CD44 expression was restricted into granule neurons strongly at the adult stage. Interestingly, OPCs expressed CD44 for a very short time, and this expression was shut off during oligodendrocyte maturation. These results strongly indicate that CD44 might inhibit oligodendrocytic differentiation, yet promote differentiation of specific subtypes of neurons and astrocytes. Further functional analysis will be needed to elucidate the roles of CD44 in cell differentiation, but the results to date suggest that CD44 may have multiple roles in cerebellar development depending on the developmental stage.

## Supporting Information

Figure S1 **The expression of Sox2/GLAST and NG2/GLAST in cerebellum at P3.** **A–D:** Double immunostaining of Sox2 and GLAST in the cerebellum at P3. **D:** High magnification of C. **E–J:** Double immunostaining of NG2 and GLAST in the cerebellum at P3. **H–J:** High magnification of E–G. Nucleus was counterstained with TO-PRO-3 (blue). Scale bars, 50 µm.
(TIF)

Figure S2 **The expression of CD44 on Bergmann glia at P7.** **A–J:** Double immunostaining of CD44 and GLAST in the cerebellum at P3. **F–J:** High magnification of A–E. Asterisk showed the cell body of CD44/GLAST double-positive Bergmann glia. Nucleus was counterstained with TO-PRO-3 (blue). Scale bars, 20 µm.
(TIF)

Figure S3 **BrdU incorporation into CD44-positive cells during postnatal development.** **A1-D1:** Immmunostaining of CD44 and BrdU at P3 (A1), P7 (B1), P10 (C1) and P14 (D1). **A2–D2:** High magnification of A1-D1. **A3–D3:** Further high magnification of A2-D2. Scale bars, 50 µm.
(TIF)
