# Supplementary figures and images for: Dynamic Changes of CD44 Expression from Progenitors to Subpopulations of Astrocytes and Neurons in Developing Cerebellum
Source: PLoS One. 2013 Jan 4;8(1):e53109. doi: 10.1371/journal.pone.0053109 (PMC3537769; doi:10.1371/journal.pone.0053109)

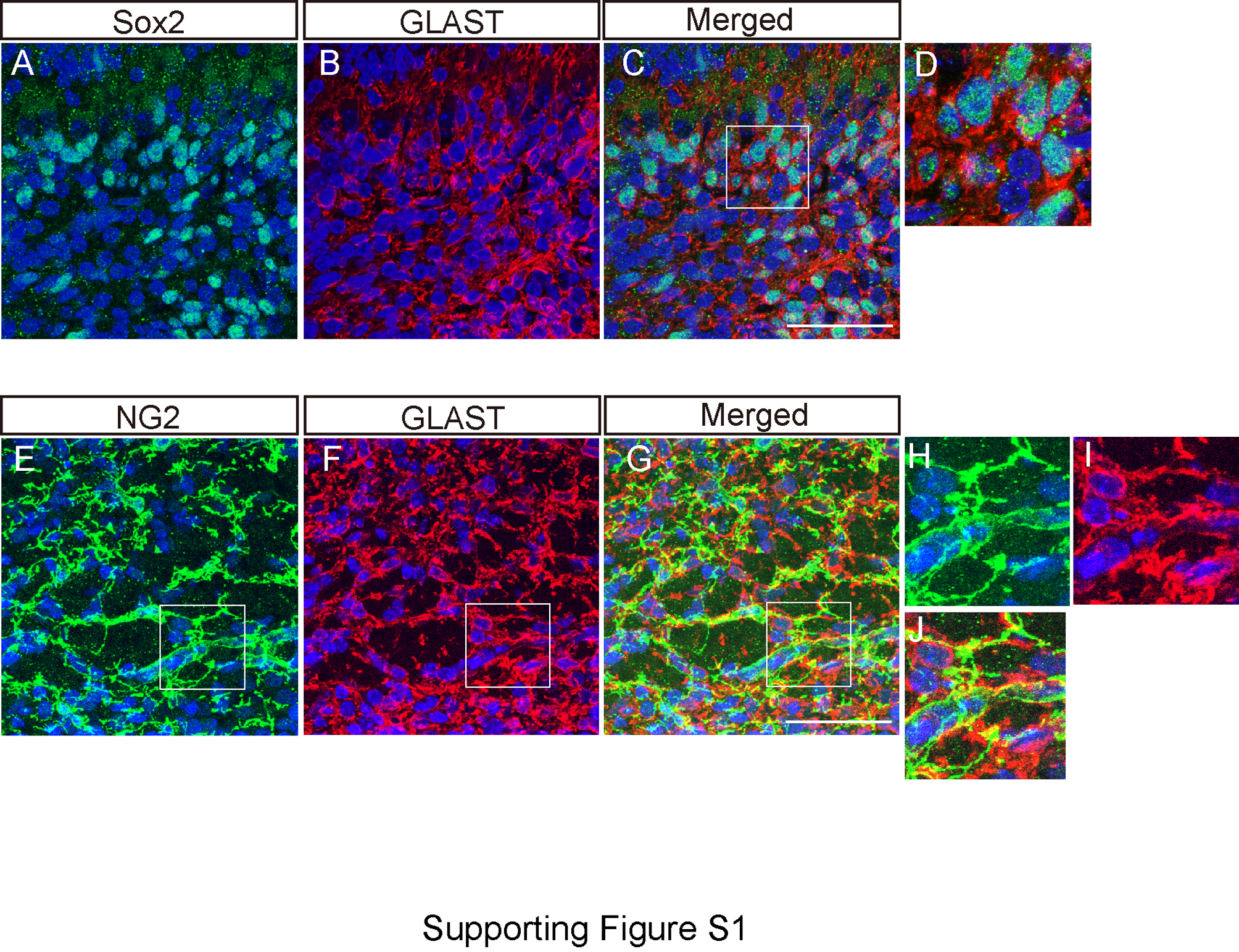

Supplement: Figure S1 — The expression of Sox2/GLAST and NG2/GLAST in cerebellum at P3. A–D: Double immunostaining of Sox2 and GLAST in the cerebellum at P3. D: High magnification of C. E–J: Double immunostaining of NG2 and GLAST in the cerebellum at P3. H–J: High magnification of E–G. Nucleus was counterstained with TO-PRO-3 (blue). Scale bars, 50 µm. (TIF) [file pone.0053109.s001.tif]

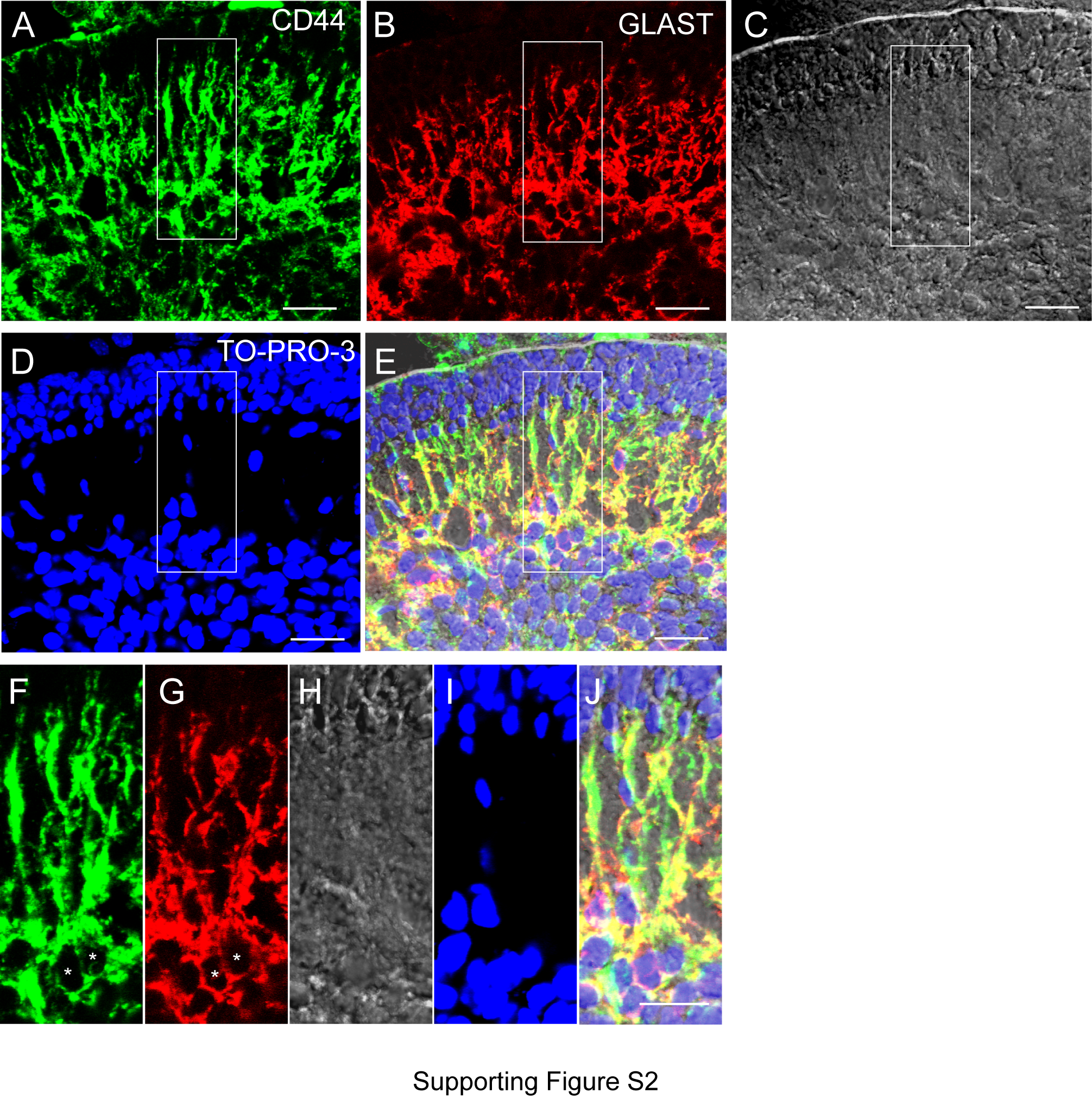

Supplement: Figure S2 — The expression of CD44 on Bergmann glia at P7. A–J: Double immunostaining of CD44 and GLAST in the cerebellum at P3. F–J: High magnification of A–E. Asterisk showed the cell body of CD44/GLAST double-positive Bergmann glia. Nucleus was counterstained with TO-PRO-3 (blue). Scale bars, 20 µm. (TIF) [file pone.0053109.s002.tif]

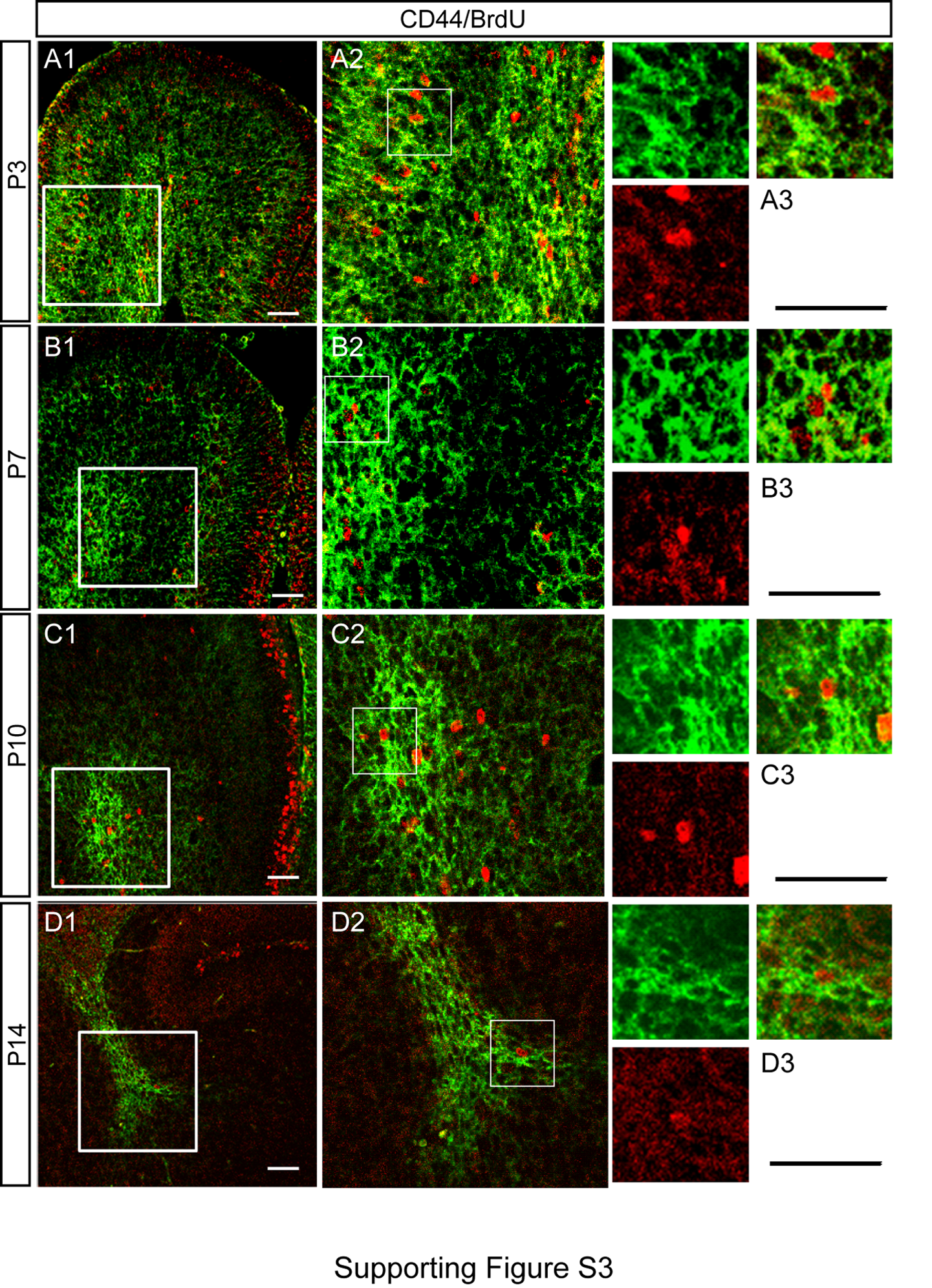

Supplement: Figure S3 — BrdU incorporation into CD44-positive cells during postnatal development. A1-D1: Immmunostaining of CD44 and BrdU at P3 (A1), P7 (B1), P10 (C1) and P14 (D1). A2–D2: High magnification of A1-D1. A3–D3: Further high magnification of A2-D2. Scale bars, 50 µm. (TIF) [file pone.0053109.s003.tif]
